# The Effect of Maternal Overweight/Obesity on Serum and Breastmilk Leptin, and Its Associations with Body Composition, Cardiometabolic Health Indices, and Maternal Diet: The BLOOM Study

**DOI:** 10.3390/metabo14040221

**Published:** 2024-04-13

**Authors:** Monika A. Zielinska-Pukos, Łukasz Kopiasz, Jadwiga Hamulka

**Affiliations:** 1 Department of Human Nutrition, Institute of Human Nutrition Sciences, Warsaw University of Life Sciences (SGGW-WULS), Nowoursynowska St. 159c, 02-776 Warsaw, Poland; monika_zielinska_pukos@sggw.edu.pl; 2 Department of Dietetics, Institute of Human Nutrition Sciences, Warsaw University of Life Sciences (SGGW-WULS), Nowoursynowska St. 159c, 02-776 Warsaw, Poland; lukasz_kopiasz@sggw.edu.pl

**Keywords:** atherogenic index of plasma (AIP), body mass index (BMI), energy value, fructose, lactation, leptin, lipid accumulation product (LAP), lipid profile, mediterranean diet, visceral adiposity index (VAI)

## Abstract

In overweight and obese patients, elevated serum and breastmilk leptin concentrations are observed, with serum leptin also being likely affected by the diet. We analyzed serum and breastmilk leptin in normal weight (NW) and overweight/obese (OW/OB) mothers, and evaluated its associations with (1) maternal anthropometric parameters; (2) markers of cardiometabolic health; and (3) the maternal diet. The BLOOM (Breastmilk and the Link to Overweight/Obesity and Maternal diet) study was conducted among 40 women (*n* = 20 OW/OB; *n* = 20, NW) who were exclusively or predominantly breastfeeding for 15.5 ± 1.2 (OW/OB group (0.99)) weeks. We collected 24 h breastmilk and fasting blood samples for leptin analysis by ELISA. Maternal dietary habits were evaluated using a 3-day dietary record and food frequency questionnaire, which were used to calculate the Polish-adapted Mediterranean Diet score. Maternal anthropometric measurements and DEXA scans were performed, and anthropometric and cardiometabolic indices were calculated. The OW mothers had 1.4 times higher serum levels, while OB mothers had 4.5 and 6.2 higher serum and breastmilk leptin levels, respectively, in comparison to the NW mothers. The FM% was correlated with serum and breastmilk leptin levels (r = 0.878, r = 0.638). Serum leptin was associated with markers of cardiometabolic health such as AIP, CMI, and VAI in the NW mothers, and with LAP in the OW/OB mothers. Higher energy, fructose intake and adherence to the Mediterranean diet were associated with serum leptin in the NW mothers (β = 0.323, 0.039–0.608; β = 0.318, 0.065–0.572; β = 0.279, 0.031–0.528); meanwhile, higher adherence to the Mediterranean diet could protect against elevated breastmilk leptin concentrations in OW/OB mothers (β = −0.444, −0.839–−0.050), even after adjustment for FM%. Our results suggest a potential association between maternal serum leptin concentrations and cardiometabolic health. In addition, we confirm the importance of healthy dietary patterns in the improvement of breastmilk composition.

## 1. Introduction

Breastfeeding is a gold standard in infant nutrition because of the various health and developmental benefits associated with the unique composition of breastmilk [1,2]. Breastmilk contains highly bioavailable nutrients and a variety of bioactive factors including leukocytes, stem cells, immunoglobins, beneficial microbes, hormones, and growth factors. In addition, the concentrations and ratios of these compounds exhibit diurnal variation, change dynamically throughout lactation, and are unique to each infant–mother dyad [1,3,4,5]. Among the environmental, parental, and infant-related factors that may contribute to the variation in breastmilk composition [3,4], the maternal health status and lifestyle, including diet and obesity, are some of the most widely discussed [4].

Currently, one-third of the global population is overweight or obese, and the prevalence has doubled since 1980 [6]. Thus, obesity-related diseases are an increasing public health burden, particularly due to non-communicable chronic diseases and their adverse effects on reproductive health [7,8]. In OW/OB mothers, lower rates of breastfeeding initiation and exclusivity, as well as a shorter duration of breastfeeding, have been observed [4]. Obesity and elevated adiposity also alter the composition of breastmilk, in particular the concentration of adipokines, the antioxidant profile, and inflammatory properties [4,9,10]. Adipokines, including leptin or adiponectin, are hormones produced by adipose tissue. OW/OB individuals secrete more leptin, which subsequently leads to the development of leptin resistance [10,11]. Leptin has a pleiotropic activity as it is involved in the regulation of energy homeostasis, food intake, and appetite, as well as fat and glucose metabolism [12]. On the other hand, alterations in the release of leptin and other cytokines (e.g., tumor necrosis factor-α (TNF-α), interleukin-6 (IL-6), and other adipocytokines) by adipose tissue could cause homeostasis imbalance, reduce insulin sensitivity, and increase inflammation and contractility. These metabolic changes play a critical role in the development and progression of many non-communicable diseases [13]. In consequence, leptin is used as a biomarker of systemic inflammation and cardiovascular disease (CVD) risk [13,14]. Up to this day, leptin has been associated with the increased risk of CVD or metabolic diseases, some types of cancer, and depression [8,11,12,15]. Contrary, it also appears to have a protective effect on bone health [16], while breastmilk leptin specifically could further be one of the key regulators of nutritional programming [17,18]. Previous studies have shown that not only obesity but also diet could positively or negatively influence leptin secretion [19].

Therefore, this study aimed to analyze the serum and breastmilk leptin levels among normal weight (NW) and overweight/obese (OW/OB) mothers, and explore the association with (1) maternal anthropometric parameters; (2) markers of cardiometabolic health; and (3) the maternal diet.

## 2. Materials and Methods

### 2.1. Study Group

**Figure 1:**
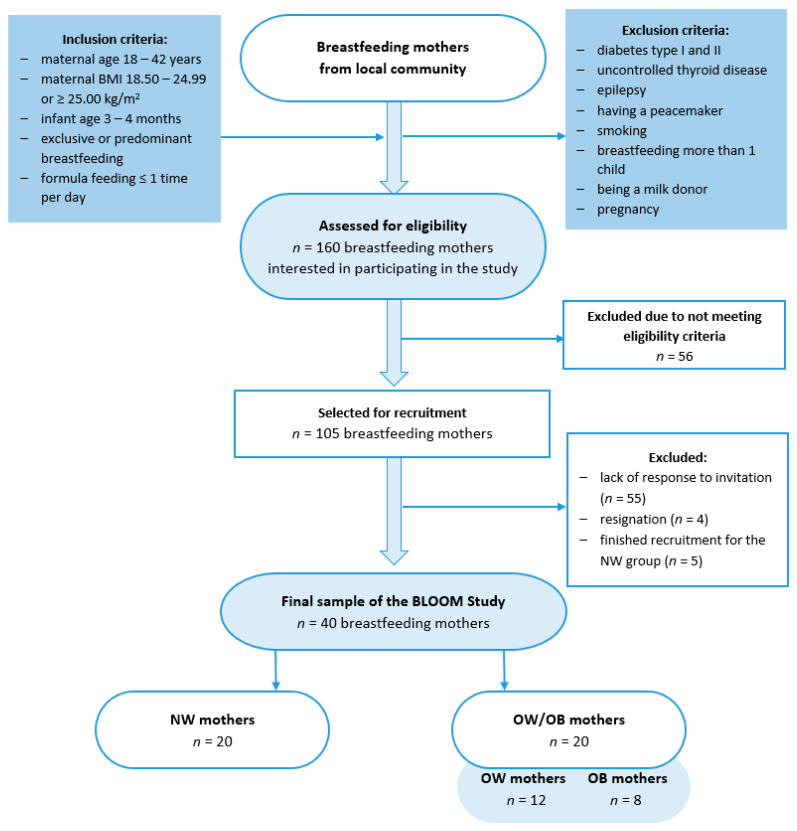
Flowchart of the study group selection. BLOOM, Breastmilk and the Link to Overweight/Obesity and Maternal diet); NW, normal weight; OW/OB, overweight or obese.

This case–control BLOOM (Breastmilk and the Link to Overweight/Obesity and Maternal diet) study was conducted between October 2022 and April 2023 among 40 mothers who exclusively or predominantly breastfed for 15.5 ± 1.2 weeks. The study group was divided into two groups: those with a normal body mass index (BMI, 18.50–24.99, kg/m^2^; NW group) and those with overweight or obesity (BMI, ≥25.00, kg/m^2^; OW/OB), according to the WHO criteria [20]. Participants were recruited through convenience sampling from the local community using social media groups, and were selected based on inclusion and exclusion criteria (Figure 1). The study was conducted in accordance with the Helsinki Declaration and was approved by the Ethics Committee of the Faculty of Human Nutrition and Consumer Sciences, Warsaw University of Life Sciences, Poland (Resolution No. 53/2021, 20 December 2021).

Based on the breastmilk leptin levels reported in the studies by Lemas et al. [21] and De Luca et al. [22], we calculated a minimum sample size using the G*Power Software 3.1.9.7 [23]. To achieve statistically significant results (80% power, significance level at 0.05), we needed to include 9 or 16 mothers in each group (n = 18 or n = 32 in total). We decided to recruit a higher number of participants, and in case of any data loss, we recruited twenty mothers for each group.

### 2.2. Breastmilk Collection

Within 24 h before the study visits, mothers were asked to collect breastmilk samples according to the instructions, which were designed to minimize diurnal and intra-feeding [5] variations in the breastmilk composition. Before and after one selected feeding from each of the four time periods (6:00–12:00; 12:00–18:00; 18:00–24:00; 24:00–06:00), mothers expressed an equal volume of fore- and hindmilk into one polypropylene bottle. The sample was stored and transported to the laboratory under refrigerated conditions and protected from light. In the laboratory, the samples were mixed in a Vortex shaker IKA MS2 (IKA Works Inc., Wilmington, NC, USA) for one minute, transferred into 2 mL Eppendorf tubes, and stored at −80 °C for further analysis.

### 2.3. Blood Collection

Fasting blood samples were collected from the ulnar vein between 7:00 a.m. and 10:00 a.m. on the day of the study visit using standard techniques. The blood samples for further analysis were centrifuged at 8000 rpm for 10 min at 4 °C and serum was stored at −80 °C. 

### 2.4. Biochemical Analysis

Biochemical analysis of the lipid profile (total cholesterol (CHOL), HDL cholesterol (HDL-C), LDL cholesterol (LDL-C), triglycerides (TG)) was performed by the certified laboratory using standard methods. Meanwhile, the LDL-C was calculated according to the Friedewald formula [24].

The serum and skim breastmilk leptin concentrations were measured by the immunoenzymatic ELISA test using a commercially available Human Leptin Quantikine ELISA Kit (R&D Systems, Minneapolis, MN, USA), according to the manufacturer’s instructions. Prior to analysis, breastmilk samples were centrifuged at 2000 rpm for 20 min at 4 °C to obtain skimmed milk. 

### 2.5. Anthropometric Assessment

Maternal anthropometric measurements (body weight and height, waist and hip circumference) were measured twice by a well-trained researcher, according to the International Society for Advancement of Kinanthropometry (ISAK) International Standards for Anthropometric Assessment guidelines [25]. We also asked mothers about their pre-pregnancy body weight. Based on these data, we calculated their pre-pregnancy and current BMI and interpreted it according to the WHO criteria [20]. The maternal body composition (fat mass (FM) and fat-free mass (FFM)) was measured using a dual-energy X-ray absorptiometer (DXA; Lunar Prodigy, GE HealthCare, Chicago, IL, USA), according to the manufacturer’s instructions. 

### 2.6. Cardiometabolic Health Indices

Based on the collected anthropometric and lipid profile data, we calculated several indices of cardiometabolic health: the atherogenic index of plasma (AIP), cardiometabolic index (CMI), lipid accumulation product (LAP), and visceral adiposity index (VAI) using the following formulas [26,27]:

AIP = log (TG/HDL-C);

CMI = (TG/HDL) × WHtR;

LAP = (WC − 58) × TG (mmo/L);

VAI = WC/(36.58 + (1.89 × BMI)) × TG/0.81 × 1.52/HDL-C.

### 2.7. Dietary Assessment

Maternal dietary habits were assessed using a food frequency questionnaire (FFQ) and 3-day dietary record methods. The FFQ was adapted from the validated Dietary Habits and Nutrition Beliefs Questionnaire KomPAN [28,29]. It included a list of 61 food items with six categories referring to the frequency of their consumption in the previous year (‘never or almost never’; ‘1–3 times a month’; ‘once a week’; ‘few times a week’; ‘once a day’; ‘few times a day’). More details can be found In the manual of the questionnaire [29]. The 3-day dietary record included the mothers’ food intake for the three typical days prior to the study visit and blood collection, with the last day being a day of breastmilk collection. Before completing the records, mothers were given detailed instructions and were asked to use typical household measures or kitchen scales to record the weight of portions consumed. The obtained records were verified by a trained dietitian, while the energy value and nutrient intake were analyzed using Dieta 6.0 Software (National Institute of Public Health NIH—National Research Institute, Warsaw, Poland). Based on the FFQ, we calculated the Polish-adapted Mediterranean Diet (Polish-aMED) [30] score, with our adaptation (in the category ‘red and processed meat’, we also included processed meat substitutes, whose availability on the Polish market has increased within recent years).

### 2.8. Statistical Analysis

All the statistical analyses were performed using STATISTICA 13.3 Software (TIBCO Software Inc., Paolo Alto, CA, USA). The normality of distribution was checked using the Shapiro–Wilk test and, when necessary, variables were log-transformed to obtain the normal distribution. Quantitative variables were presented as mean (M) ± standard deviation (SD) or median (upper–lower quartile) for variables with a non-normal distribution, and qualitative data were presented as frequencies (n (%)). Differences between the NW and OW/OB groups were assessed using the t-Student test or U-Mann–Whitney test for quantitative variables and Fisher’s exact test for qualitative variables. Differences between the NW, OW, and OB groups were assessed using ANOVA followed by the RIR Tukey post hoc test, or by the ANOVA Kruskal–Wallis test followed by a post hoc test for quantitative variables and a chi2 test for qualitative variables. We examined the correlations between leptin and the mothers’ anthropometric parameters, lipid profile, and cardiometabolic indices using Pearson or Spearman rank correlations.

Furthermore, stepwise forward multiple regression analysis was performed to identify the best dietary and anthropometric predictors of serum and breastmilk leptin. Based on the performed analysis, we selected three significant dietary predictors (energy value (kcal/d), fructose intake (g/d), and Pl-aMED score) and one anthropometric predictor (FM%). Then, we created multivariate linear models adjusted for maternal age. Analyses were performed on log-transformed variables and separately for the total, NW, and OW/OB groups. Afterward, we conducted the post hoc power analysis for created regression models using the G*Power Software 3.1.9.7 [23].

## 3. Results

The characteristics of the participants are shown in Table 1. There were no significant differences between the NW and OW/OB groups in maternal characteristics, gestational weight gain, CHOL, LDL-C, AIP, and dietary variables.

**Figure 2:**
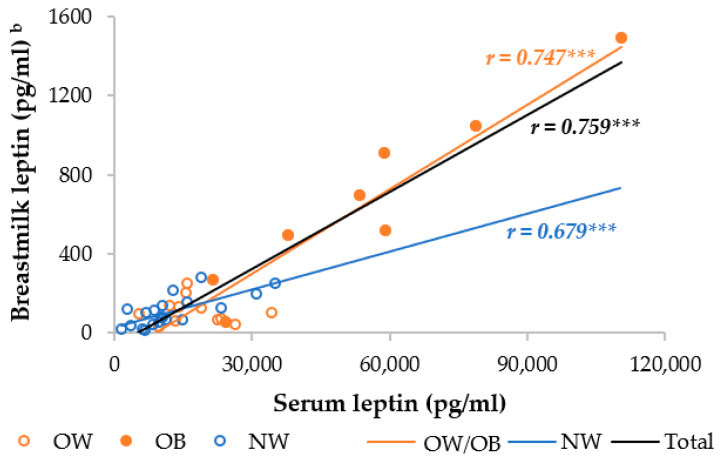
Correlation between breastmilk leptin and serum leptin among NW and OW/OB mothers. NW, normal weight; OW/OB, overweight or obese. ^b^ The result of the Pearson correlations conducted on log-transformed data. *** *p* ≤ 0.001.

The OW/OB group had a significantly higher serum and skimmed breastmilk leptin level compared to the NW group. In the separate analysis, the OB mothers had, respectively, 4.5- and 3.2-fold higher serum leptin levels than the NW and OW mothers, whereas the OW mothers had an insignificantly 1.4-fold higher serum leptin level (Table S1). The OW and NW mothers had similar breastmilk leptin levels, but the OB mothers had 6.2 times higher breastmilk levels than the NW and OW mothers. Moreover, the serum and breastmilk leptin levels were significantly different between the OB and both the OW and NW mothers, but not between the OW and NW mothers. We observed a strong correlation between serum and breastmilk leptin, which was higher in the OW/OB group compared to the NW group (Figure 2).

**Figure 3:**
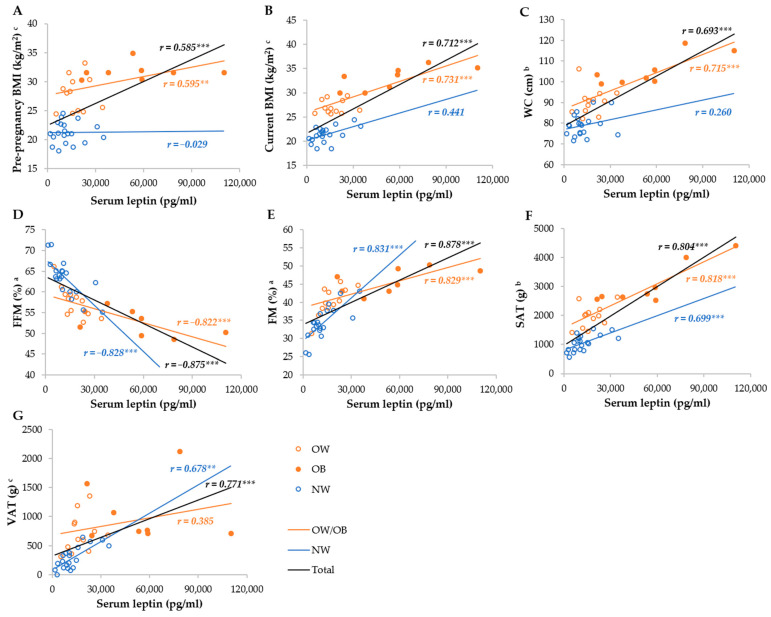
Correlation between serum leptin and anthropometric parameters among NW and OW/OB mothers. BMI, body mass index; FFM, fat-free mass; FM, fat mass; NW, normal weight; OW/OB, overweight or obese; SAT, subcutaneous adipose tissue; WC, waist circumference; VAT, visceral adipose tissue. ^a^ Result of the Pearson test conducted on original data; ^b^ result of the Pearson correlations conducted on log-transformed data; ^c^ results of the Spearman rank correlations. ** *p* ≤ 0.01; *** *p* ≤ 0.001. (**A**) Correlation between serum leptin and pre-pregnancy BMI, (**B**) correlation between serum leptin and current BMI, (**C**) correlation between serum leptin and WC, (**D**) correlation between serum leptin and FFM, (**E**) correlation between serum leptin and FM, (**F**) correlation between serum leptin and SAT, and (**G**) correlation between serum leptin and VAT.

**Figure 4:**
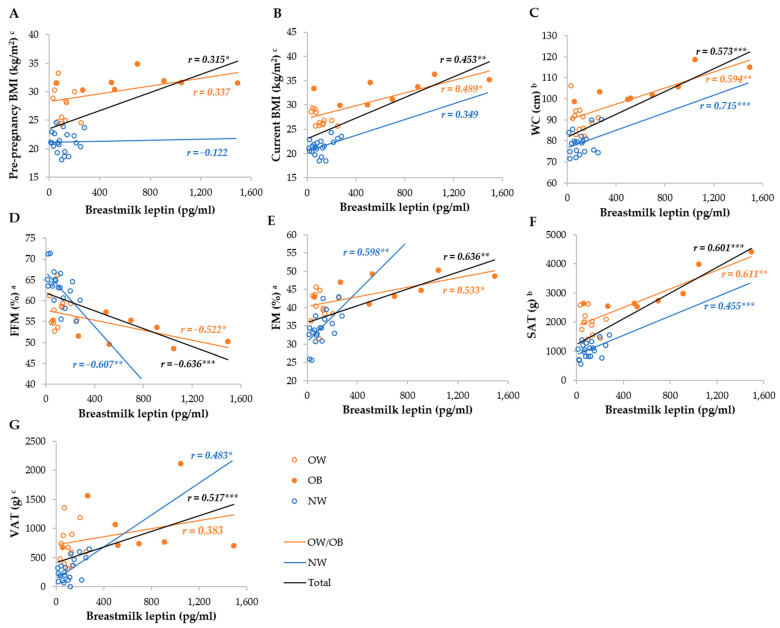
Correlation between breastmilk leptin and anthropometric parameters among NW and OW/OB mothers. BMI, body mass index; FFM, fat-free mass; FM, fat mass; NW, normal weight; OW/OB, overweight or obese; SAT, subcutaneous adipose tissue; WC, waist circumference; VAT, visceral adipose tissue. ^a^ Result of the Pearson test conducted on original data; ^b^ result of the Pearson correlations conducted on log-transformed data; ^c^ results of the Spearman rank correlations. * *p* ≤ 0.05; ** *p* ≤ 0.01; *** *p* ≤ 0.001. (**A**) Correlation between breastmilk leptin and pre-pregnancy BMI, (**B**) correlation between breastmilk leptin and current BMI, (**C**) correlation between breastmilk leptin and WC, (**D**) correlation between breastmilk leptin and FI (**E**) correlation between breastmilk leptin and FM, (**F**) correlation between breastmilk leptin and SAT, and (**G**) correlation between breastmilk leptin and VAT.

Serum leptin was most strongly correlated with the FM%, FFM%, SAT, VAT, WC, current, and pre-pregnancy BMI in the total group, whereas it was not correlated with the BMI and WC, as well as VAT, in the NW and OW/OB groups, respectively (Figure 3). Breastmilk leptin was less correlated with anthropometric parameters than serum leptin, and the strongest correlations were observed with FM%, FFM%, SAT, WC, and VAT, whereas they were not correlated with BMI in the NW group, and VAT and pre-pregnancy BMI in the OW/OB group. We observed stronger correlations between SAT and serum and breastmilk leptin in the OW/OB group, and with VAT, FM%, and FFM% in the NW group (Figure 4).

We observed that serum leptin was negatively correlated with CHOL (NW group) and HDL-C (total and NW groups). At the same time, it was positively correlated with TG (total group), AIP, CMI, VAI (total and NW groups), and LAP (total and NW groups; Table 2). We observed similar correlation coefficients between breastmilk leptin and cardiometabolic indices.

Multivariate linear regression analysis showed that FM% was a strong and significant predictor of both serum and breastmilk leptin (Table 3). In addition, we found that in the NW, but not in the total and OW groups, the serum leptin concentration increased with the dietary energy value (β = 0.323, 95% CI 0.039–0.608) and fructose intake (β = 0.318, 95% CI 0.065–0.572), and increased with higher adherence to the Mediterranean diet (β = 0.279, 95% CI −0.031–0.528); however, in the NW group, the Pl-aMED score was moderately correlated with energy intake (r = 0.352, *p* = 0.128 vs. r = 0.042, *p* = 0.860 in the OW/OB), which may explain the observed association. In the OW/OB mothers, we observed that a higher adherence to the Mediterranean diet was associated with lower breastmilk leptin (β = −0.444, 95% CI −0.839–−0.050). However, the power of this analysis is slightly below the recommended value.

## 4. Discussion

In the present study, we investigated the associations between serum and skimmed breastmilk leptin, maternal anthropometric and cardiometabolic health indices, and diet in the NW and OW/OB mothers. Our results confirm that overweight/obesity is associated with elevated breastmilk and serum leptin, and that these values are highly correlated. However, in the separate analysis of the OW/OB group, we found that obesity influences the leptin concentration greater than overweight. We also found that body composition was more strongly correlated with serum and breastmilk leptin concentrations, whereas the effect of fat distribution was different in the NW and OW/OB mothers. In addition, serum leptin was associated with markers of increased cardiovascular disease risk, especially in the NW mothers. Last but not least, we observed that higher dietary energy and fructose intake were associated with higher serum leptin in the NW mothers, whereas a higher adherence to the Mediterranean diet may protect against elevated breastmilk leptin in the OW/OB mothers.

### 4.1. Serum and Breastmilk Leptin and Maternal Anthropometrics

The OW/OB mothers in our study had 1.8- and 3.1-fold higher serum and breastmilk leptin concentrations, respectively, than the NW mothers. However, in the separate analysis, we found that the OB mothers had, respectively, 4.5- and 3.2-fold higher serum leptin levels than the NW and OW mothers, whereas the difference between the OW and NW mothers was insignificant. The OW and NW mothers had similar breastmilk leptin levels, but the OB mothers had 6.2 times higher breastmilk levels than both the NW and OW mothers. It is well known that overweight/obesity and/or increased adiposity are associated with elevated serum leptin due to increased adipocyte secretion and leptin resistance [11,12]. At this point, it should be noted that not all leptin circulates free, and some is bound to the circulating soluble leptin receptor (LepRe). Leptin bound to LepRe is Inactive and inaccessible to other leptin membrane receptors. In lean individuals, up to about 65% of leptin circulates bound to this receptor, while in obese individuals, only 15% is bound and 85% is free leptin [31,32]. Studies conducted during lactation have also shown higher serum [33] and breastmilk leptin concentrations in overweight or obese mothers [10,21,22,33,34,35]. This is due to strong correlations between serum and breastmilk leptin [33,36,37,38], which were also confirmed in our study. This indicates that leptin is transferred from serum to breastmilk [36,37,38]. However, some amount of leptin could also be synthesized by mammary epithelial cells and secreted into the breastmilk in milk fat globules [39,40]. In addition, it has been suggested that adipocytes and leptin could influence mammary epithelial cell proliferation, differentiation, and/or apoptosis, which can contribute to increased leptin concentrations in breastmilk [10,40]. This may also explain why we observed stronger correlations between breastmilk leptin concentrations and body composition than between BMI or WC, which are not direct measures of adiposity. Moreover, the higher transfer of leptin into breastmilk in obese women may also be explained by the predominance of the active free form of circulating leptin in their serum. We observed slightly stronger correlations between serum leptin, BMI (pre-pregnancy and current), and WC, but they were lower than with FM%, VAT, and SAT. Moreover, in both cases, the correlations with BMI and WC were not significant in the NW mothers, probably due to their lower adiposity. On the contrary, it has been previously suggested that leaner mothers could have a greater sensitivity to adiposity [41]. Nevertheless, our results are consistent with the majority of previous studies that found moderate or no correlations between the current maternal or pre-pregnancy BMI and breastmilk leptin [9,10,22,41,42,43], and that FM% (if assessed) was a stronger predictor of breastmilk leptin compared to BMI [41,44,45]. We observed intriguing results regarding the association between adipose tissue distribution and serum or breastmilk leptin. In our group, SAT was more strongly correlated with the serum and breastmilk leptin concentration (especially in the OW/OB group), compared to VAT (results in the OW/OB group were insignificant). To the best of our knowledge, this is the first study to report the association between the serum and breastmilk leptin concentration in NW and OW/OB mothers. However, these results are not surprising given that in women, leptin secretion rates can be two to three times higher in SAT than in VAT, independent of obesity or menopausal status [8,46].

### 4.2. Maternal Leptin and Cardiometabolic Health

Leptin has pro-inflammatory properties and is one of the mediators of systemic inflammation [11]. Increased leptin secretion causes adipose hypertrophy, insulin resistance, dyslipidemia, hypertension, and thickness of the common carotid artery [8,47]. Therefore, elevated leptin levels could increase the risk of the subsequent development of obesity-related non-communicable diseases such as CVD, type 2 diabetes, metabolic syndrome (MetS), and even some types of cancer [8,12,47,48]. In this study, we examined the associations between serum and breastmilk leptin, the maternal lipid profile, and cardiometabolic health indices, such as AIP, CMI, LAP, and VAI. These indices are based on anthropometric data (WC, BMI, WHtR) and/or lipid profile (HDL, TG), and could be useful for the early diagnosis or identification of patients with diabetes, CVD, and MetS [26,27,49].

In our study, we observed that serum leptin was negatively correlated with HDL-C (Total and NW groups) and positively correlated with TG (only in the Total group), AIP, CMI, VAI (Total and NW groups), and LAP (Total and OW/OB groups). Therefore, our results indicate that these associations are dependent of obesity status and that leptin could be a better predictor of cardiometabolic health indices in NW mothers. Previous studies conducted in non-lactating individuals have shown that CMI and VAI are good predictors of the “metabolic obesity with normal body weight” (MONW) phenotype, which could explain the stronger and more significant associations observed in the NW group [27]. On the other hand, previous studies have associated serum leptin with an increased risk of insulin resistance in children [47] and MetS in adults [11], independent of obesity status. However, both studies included larger numbers of participants, analyzed more metabolic biomarkers (e.g., fasting glucose and insulin), and were conducted in non-lactating women. It is well known that significant changes in lipid and glucose metabolism occur during pregnancy, manifested by physiologically elevated CHOL-C, LDL-C and TG, insulin resistance, and glucose intolerance [50]. Breastfeeding is associated with improvements in these biomarkers; CHOL-C and TG decrease from 2 to 6 months of lactation and return to baseline after one year of lactation [50]. Thus, breastfeeding is associated with a longitudinal decrease in the cardiometabolic risk in mothers, even in the post-menopausal period [50]. To the best of our knowledge, this was the first study to evaluate these indices in lactating individuals, so it is unclear how these indicators adjust for this physiological state. However, in light of the above, our results seem to be logical. Therefore, prospective studies are needed to determine the associations between maternal overweight/obesity and mothers’ metabolic profile, breastfeeding status, and subsequent metabolic health.

### 4.3. Serum and Breastmilk Leptin—Associations with Maternal Diet

The results of the present study showed that the energy value and fructose intake during the 72 h before blood collection were significant predictors of serum leptin in the NW mothers, even after adjustment for FM%. The increase in the serum leptin concentration associated with the higher energy value of the diet in the previous days is not surprising, as leptin is a key regulator of energy balance, food intake, and appetite [12]. We assume that the increase in serum leptin following a more energetic diet was a metabolic response leading to a subsequent decrease in food intake. This would be supported by the results of several animal and human studies showing that leptin administration reduces food and calorie intake [12,51]. In addition, a recent analysis of intervention studies conducted in lean individuals showed that serum leptin levels directly before a meal are inversely correlated with caloric intake [51]. Another intervention conducted in adult men showed that a decrease in leptin during energy restriction was associated with an increase in appetite, but due to sex differences, a smaller association would be expected in women [52]. To date, only Leghi et al. [53] have investigated a dietary intervention in breastfeeding mothers. They showed that a 2-week energy restriction resulted in a decrease in breastmilk leptin, adiponectin, and insulin, which is in line with previous studies assessing serum leptin in non-lactating women. In our study, we did not observe a significant association between maternal energy intake and breastmilk composition. This could be a consequence of the fact that our participants followed a habitual diet and the associations were too weak to be observed. The association between habitual dietary intake and serum leptin concentrations has received little attention, and the studies that have been conducted have obtained less consistent results than intervention studies. Two studies conducted in postmenopausal women [54] and obese adults aged 40–59 years [55] showed inverse associations between the serum leptin concentration and energy intake, and another study found no significant association [56]. The authors also concluded that their results support the role of leptin in the physiological regulation of food intake. Although these studies also observed associations with other dietary components, such as carbohydrates [54], total fat [54], saturated fatty acids [54], protein [35], and dietary fiber [56], we did not confirm this association in our study. On the other hand, we observed that fructose intake was positively associated with serum leptin in the NW mothers. This is in line with the previous animal studies linking high-fat and high-fructose diets with the development of hyperleptinemia [19]. A similar effect was observed in humans, where 4-week fructose supplementation led to a continuous rise in the fasting leptin concentration [57].

One of the most important findings of our study was that the Mediterranean diet may help to limit increases in the breastmilk leptin concentration in OW/OB mothers. On the other hand, in the NW mothers, it was related to higher serum leptin. However, in this group, Pl-aMED was moderately correlated with energy intake, which may explain the observed association. Despite the power of the analysis conducted in the OW/OB group being 8% lower than the recommended 80%, the observed results are indeed in line with the literature [14,58,59]. The traditional Mediterranean diet is characterized by a higher consumption of vegetables and fruits, legumes, whole grains, nuts and seeds, olive oil, fish, and seafood, while minimizing the intake of meat, ultra-processed foods, and alcohol [60]. Previous studies found that the Mediterranean diet is associated with lower serum inflammatory biomarkers including leptin [14,58,59]. On the other hand, some studies have not confirmed this [61], or showed that the beneficial effect was only in participants with weight reduction [62]. Additionally, the Mediterranean diet has anti-inflammatory properties, and improves the lipid profile, blood pressure, and insulin sensitivity [61]. Thanks to these effects, it plays a beneficial role in the prevention of non-communicable diseases and mortality, as well as improves overall health and well-being [14,30,61]. However, little is known about the association between the Mediterranean diet and leptin during pregnancy and lactation. Interestingly, an intervention study conducted during pregnancy showed lower gestational weight gain, birthweight, neonate fat mass (%), and cord leptin in the Mediterranean intervention group [58]. To the best of our knowledge, this is the first study to investigate the associations between adherence to the Mediterranean diet and the serum and breastmilk leptin concentration in lactating mothers. However, several studies have investigated the association with breastmilk composition or various health outcomes during lactation. The Mediterranean diet during lactation could have a beneficial effect on the fatty acid profile [60], total antioxidant content [63], and selenium [64], calcium and zinc [65] content of breastmilk. Tabasso et al. [66], in a study conducted among lactating mothers in Italy, found that a higher adherence to the Mediterranean diet was associated with lower adipose tissue deposition during breastfeeding (without differences in body weight or BMI). This observation could partially explain the association between Pl-aMED and leptin observed in our study. However, because the results remained significant after adjustment for FM%, we can assume that this goes beyond the association with body composition and is related to an anti-inflammatory effect of this diet. This is supported by the results of Stendell-Hollis [67], who found that a 4-month Mediterranean-style dietary intervention improved body mass reduction, and decreased the concentration of serum inflammatory biomarkers such as TNF-α, but not IL-6, in overweight breastfeeding mothers. It was also shown that higher adherence to the Mediterranean diet could reduce the risk of postpartum depression [68]. Interestingly, this may also be related to leptin, as it has been suggested that leptin insufficiency and/or resistance may contribute to the development of depression [15].

### 4.4. Possible Implications to the Infant Health

In recent years, much interest has focused on the effect of leptin on nutritional programming [17,18]. Previous animal and human studies have shown that during infancy, ingested leptin is absorbed into the circulation and exerts biological effects [38,69]. With that said, leptin could beneficially influence the development of the intestinal microbiota [21]. But the effect of leptin on infant growth and body composition has been more widely discussed with ambiguous results [2,18,43]. A recent systematic review revealed that leptin showed consistent negative associations with infant anthropometrics (weight, weight gain, length, BMI z-score, FM%). However, the authors highlighted that most of the studies did not adequately control for confounders, including maternal BMI [2]. We, and other authors [9,21,70], have shown that infants born to OW/OB mothers are exposed to a few times higher levels of breastmilk leptin than those born to NW mothers. It has been hypothesized that exposure to elevated leptin levels could lead to the development of leptin resistance, subsequent alterations in appetite regulation and an increased risk of obesity [38]. Maternal obesity is an important factor that increases the risk of childhood obesity [70,71], but at the same time, breastfeeding plays a crucial protective role against the development of obesity. Having said that, a limited number of studies have compared the effects of leptin programming between NW and OW/OB mothers. It has been shown that maternal OW/OB could alter the infant intestinal microbiota [21] and breastmilk supply of leptin-related miRNAs, which may influence infant development [70]. Additionally, more impact than leptin concentration could have a total 24-h infant leptin intake via breastmilk [72,73]. Therefore, further studies should understand the role of breastmilk from OW/OB mothers in nutritional programming [18].

### 4.5. Strengths and Limitations

This study has several strengths. Firstly, our participants were matched for socio-economic characteristics and at a similar period of lactation, which reduced the possible influence of covariates. Secondly, we collected both serum and breastmilk samples, which allowed us to examine the associations more widely. Thirdly, we collected 24 h breastmilk samples including fore- and hindmilk, which reduced the interference of intra-feeding and diurnal variability in leptin concentrations [74]. Fourthly, we assessed the maternal body composition using DEXA, a “gold standard” in the assessment of body composition [27,75]. Fifthly, we assessed the maternal dietary habits using the FFQ and 3-day dietary records. Nevertheless, our study also has some limitations. Firstly, we analyzed leptin in skimmed breastmilk, but it has been shown that leptin concentrations are higher in whole milk [36,45]. Yet, despite this, the majority of studies have investigated leptin in skimmed milk [18]. Secondly, our results revealed a high variability among the OW/OB mothers, which indicates the importance of separate analyses of OW and OB mothers. Unfortunately, in our study, 12 mothers were overweight and 8 obese, so we were unable to conduct separate analyses in those three groups. Thirdly, our study had a cross-sectional design without follow-up, which could provide more comprehensive data on maternal anthropometrics, diet, breastmilk composition, and cardiometabolic health, as well as leptin variability and its determinants. Fourthly, our participants were voluntary, not randomly selected, so we cannot generalize the results of our study to the total population of breastfeeding mothers. Moreover, our results could be affected by selection bias, as volunteers may have different characteristics and lifestyle-related behaviors (e.g., higher physical activity, healthy dietary patterns, or longer duration of breastfeeding) than the targeted population. Fifthly, the results of the linear regression in subgroups were sometimes underpowered, so a larger study group is needed for more complex statistical analysis (e.g., 34 patients in the group would be sufficient to achieve 80% of power in the model with breastmilk leptin as the dependent variable). Sixthly, we did not include infants in our study, so we cannot evaluate the influence of maternal overweight/obesity through breastmilk composition on their health and development.

## 5. Conclusions

The current findings add to a growing body of evidence on the influence of maternal overweight/obesity on breastmilk composition. We found that OW/OB mothers had significantly higher serum and breast milk leptin concentrations, with a greater effect in obese mothers. We also showed that serum leptin was significantly correlated with cardiometabolic health indices (especially in the NW mothers), which according to the literature, are predictors of the risk of developing CVD, type 2 diabetes or MetS among non-lactating individuals. However, further studies need to be performed to establish whether the serum leptin levels during lactation affect the well-known protective effect of breastfeeding on maternal cardiometabolic health. We also found that the maternal habitual dietary intake of energy and fructose had a negative effect, but that adherence to the Mediterranean diet had a positive effect on breastmilk leptin concentrations in the OW/OB mothers. Our results support the importance of healthy dietary patterns throughout lactation. Furthermore, the observed OW/OB-related differences in the analyzed associations indicate differences in the metabolic profile between NW and OW/OB mothers. So, ideally, studies should also evaluate metabolic profiles to better understand this phenomenon. Further research should longitudinally investigate the causal relationship between the maternal diet, breastmilk hormone and the metabolic profile, especially in the context of maternal obesity, subsequent cardiometabolic health, and the influence on infant growth and development. Moreover, further studies should favor a separate analysis of OW and OB, rather than clustering OW and OB patients.

## Figures and Tables

**Table 1:** Distribution of general variables of the participants among NW and OW/OB mothers.

Variable	Study Group M ± SD/Me (25–75)/*n* (%)			*p*-Value
	Total *n* = 40	NW *n* = 20	OW/OB *n* = 20	
Maternal age (years)	32.4 ± 3.9	31.9 ± 4.3	32.9 ± 3.5	0.431 ^a^
Lactation duration (weeks)	15.5 ± 1.2	15.7 ± 1.0	15.2 ± 1.3	0.219 ^a^
Breastfeeding per day	11.0 (10.0–13.5)	11.0 (10.0–13.0)	12.0 (10.5–14.0)	0.514 ^c^
Primiparous (*n* (%))	26 (65%)	14 (70%)	12 (60%)	0.741 ^d^
Anthropometric parameters				
Pre-pregnancy BMI (kg/m^2^)	24.5 (21.0–30.3)	21.0 (19.9–22.6)	30.3 (26.8–31.6)	<0.001 ^c^
Gestational weight gain (kg)	13.4 ± 5.7	13.5 ± 4.1	13.3 ± 7.0	0.924 ^a^
Current BMI (kg/m^2^)	25.0 (21.4–28.9)	21.4 (20.4–22.4)	28.9 (26.3–32.3)	<0.001 ^c^
WC (cm)	87.8 ± 11.8	79.1 ± 5.3	96.5 ± 10.0	<0.001 ^b^
FFM (%)	59.5 ± 5.7	63.3 ± 4.3	55.7 ± 4.3	<0.001 ^a^
FM (%)	38.5 ± 6.1	34.4 ± 4.5	42.6 ± 4.6	<0.001 ^a^
VAT (g)	563.6 (238.5–727.5)	284.8 (117.5–420.5)	727.5 (600.5–982.0)	<0.001 ^c^
SAT (g)	1476 (1061–2368)	1053 (824–1244)	2405 (1942–2641)	<0.001 ^c^
Lipid profile				
CHOL (mg/dL)	198.5 ± 34.1	200.0 ± 29.5	197.0 ± 38.9	0.789 ^a^
HDL-C (mg/dL)	74.5 ± 17.1	81.1 ± 13.6	67.8 ± 17.9	0.005 ^b^
LDL-C (mg/dL)	111.5 ± 32.5	107.3 ± 24.4	115.6 ± 39.1	0.427 ^a^
TG (mg/dL)	51.9 (46.7–71.2)	48.1 (44.4–60.9)	63.2 (50.2–74.6)	0.038 ^c^
Cardiometabolic indices				
AIP	−0.18 (−0.27–0.03)	−0.22 (−0.27–−0.15)	−0.02 (−0.23–0.13)	0.060 ^c^
CMI	0.15 (0.12–0.29)	0.13 (0.11–0.15)	0.23 (0.16–0.35)	0.002 ^c^
LAP	21.6 ± 14.9	13.6 ± 5.9	29.7 ± 16.9	<0.001 ^b^
VAI	0.61 (0.49–0.95)	0.52 (0.43–0.61)	0.80 (0.58–1.16)	0.010 ^c^
Dietary parameters				
Energy (kcal/d) ^a^	2152 ± 336	2094 ± 326	2211 ± 344	0.281 ^b^
Fructose (g/d) ^b^	11.0 ± 6.5	9.5 ± 4.8	12.5 ± 7.6	0.156 ^b^
Pl-aMED	5.0 (4.0–6.0)	5.0 (3.5–6.5)	5.5 (4.0–6.0)	0.945 ^c^
Leptin (pg/mL)				
Serum	22,552.3 ± 22,194.2	12,406.5 ± 8819.0	22,764.4 ± 26,769.5	<0.001 ^b^
Breastmilk	224.7 ± 310.0	108.9 ± 77.8	340.5 ± 403.7	0.015 ^b^

AIP, atherogenic index of plasma; BMI, body mass index; CHOL, total cholesterol; CMI, cardiometabolic index; FFM, fat-free mass; FM, fat mass; HDL-C, HDL cholesterol; LAP, lipid accumulation product; LDL-C, LDL cholesterol; M, mean; Me, median; NW, normal weight; OW/OB, overweight or obese; Pl-aMED, Polish-adapted Mediterranean Diet; SAT, subcutaneous adipose tissue; SD, standard deviation; TG, triglycerides; WC, waist circumference; VAI, visceral adiposity index; VAT, visceral adipose tissue. ^a^ result of the *t*-Student test conducted on original data; ^b^ result of the *t*-Student test conducted on log-transformed data; ^c^ results of the U-Mann–Whitney test; ^d^ results of Fisher’s exact test.

**Table 2:** Correlations between serum and breastmilk leptin and lipid profile, and cardiometabolic indices among NW and OW mothers.

Variable	Serum Leptin			Breastmilk Leptin		
	Total *n* = 40	NW *n* = 20	OW/OB *n* = 20	Total *n* = 40	NW *n* = 20	OW/OB *n* = 20
Lipid profile						
CHOL (mg/dL) ^a^	−0.055	−0.618 **	0.397	0.100	−0.362	0.400
HDL-C (mg/dL) ^a^	−0.468 **	−0.774 ***	−0.020	−0.230	−0.608 **	0.184
LDL-C (mg/dL) ^a^	0.079	−0.389	0.335	0.118	−0.156	0.223
TG (mg/dL) ^b^	0.414 **	0.180	0.352	0.416 **	0.277	0.439
Cardiometabolic indices						
AIP ^b^	0.451 **	0.654 **	0.259	0.341 *	0.650 **	0.202
CMI ^b^	0.578 ***	0.651 **	0.272	0.429 **	0.606 **	0.226
LAP ^a^	0.683 ***	0.428	0.601 **	0.614 ***	0.398	0.601 **
VAI ^b^	0.501 ***	0.609 **	0.209	0.402 *	0.657 **	0.188

AIP, atherogenic index of plasma; CHOL, total cholesterol; CMI, cardiometabolic index; HDL-C, HDL cholesterol; LAP, lipid accumulation product; LDL-C, LDL cholesterol; NW, normal weight; OW/OB, overweight or obese; TG, triglycerides; VAI, visceral adiposity index. ^a^ Result of the Pearson correlations conducted on log-transformed data; ^b^ results of the Spearman rank correlations conducted on original data. * *p* ≤ 0.05; ** *p* ≤ 0.01; *** *p* ≤ 0.001.

**Table 3:** Results of multivariable linear regression analysis exploring associations between serum or breastmilk leptin and selected dietary variables.

Model	Variable	Total *n* = 40		NW *n* = 20		OW/OB *n* = 20	
		β (95% CI), *p*-Value	R^2^, *p*-Value, Power	β (95% CI), *p*-Value	R^2^, *p*-Value, Power	β (95% CI), *p*-Value	R^2^, *p*-Value, Power
Serum leptin							
1	Energy (kcal/d)	0.048 (−0.117–0.213), 0.561	0.76, <0.001, 1.00	0.323 (0.039–0.608), 0.029	0.74, <0.001, 0.99	−0.094 (−0.402–0.215), 0.528	0.64, ≤0.001, 1.00
	Maternal age	−0.058 (−0.221–0.106), 0.479		−0.098 (−0.352–0.155), 0.422		−0.035 (−0.346–0.277), 0.817	
	FM%	0.890 (0.727–1.053), <0.001		0.976 (0.688–1.264), <0.001		0.814 (0.496–1.131), <0.001	
2	Fructose (g/d)	0.066 (−0.101–0.233), 0.430	0.76, <0.001, 1.00	0.318 (0.065–0.572), 0.017	0.76, <0.001, 0.99	−0.100 (−0.396–0.197), 0.486	0.64, ≤0.001
	Maternal age	−0.064 (−0.229–0.100), 0.479		−0.183 (−0.443–0.076), 0.154		−0.040 (−0.347–0.267), 0.787	
	FM%	0.870 (0.707–1.033), <0.001		0.771 (0.522–1.019), <0.001		0.842 (0.538–1.146), <0.001	
3	Pl-aMED	0.077 (−0.082–0.236), 0.331		0.279 (0.031–0.528), 0.030		−0.231 (−0.509–0.046), 0.097	
	Maternal age	−0.047 (−0.206–0.112), 0.552	0.76, <0.001,	−0.080 (−0.333–0.174), 0.515	0.74, <0.001,	−0.103 (−0.392–0.185), 0.458	0.69, ≤0.001,
	FM%	0.880 (0.721–1.039), <0.001	1.00	0.854 (0.599–1.109), <0.001	0.99	0.859 (0.576–1.142), <0.001	0.99
Breastmilk leptin							
4	Pl-aMED	−0.203 (−0.457–0.052), 0.115	0.39, ≤0.001, 0.99	−0.087 (−0.500–0.325), 0.659	0.29, 0.036, 0.55	−0.444 (−0.839–−0.050), 0.029	
	Maternal age	−0.007 (−0.262–0.248), 0.957		−0.196 (−0.617–0.224), 0.337		0.052 (−0.357–0.461), 0.791	0.37, 0.015,
	FM%	0.632 (0.377–0.887), <0.001		0.544 (0.121–0.967), 0.015		0.507 (0.105–0.909), 0.017	0.72

CI, confidence interval; FM%, percentage of fat mass; NW, normal weight; OW/OB, overweight or obese; Pl-aMED, Polish-adapted Mediterranean Diet. Analyses were conducted on log-transformed variables.

## Data Availability

The data presented in this study are openly available in RepOD, repod.icm.edu.pl at https://doi.org/10.18150/3OCJMS.

## Supplementary Materials

The following supporting information can be downloaded at: https://www.mdpi.com/article/10.3390/metabo14040221/s1, Table S1: Distribution of general variables of the participants among NW, OW and OB mothers. Figure S1. Correlation between serum leptin and anthropo-metric parameters among NW and OW/OB mothers with serum leptin not exceeding 50,000 pg/mL. Figure S2. Correlation between breastmilk leptin and anthropometric parameters among NW and OW/OB mothers with breastmilk levels not exceeding 600 pg/mL.
