# Supplementary material for: The Effect of Maternal Overweight/Obesity on Serum and Breastmilk Leptin, and Its Associations with Body Composition, Cardiometabolic Health Indices, and Maternal Diet: The BLOOM Study
Source: Metabolites. 2024 Apr 13;14(4):221. doi: 10.3390/metabo14040221 (PMC11051946; doi:10.3390/metabo14040221)
Supplement: Supplementary file 1 [file metabolites-14-00221-s001.zip › metabolites-2890516-supplementary.pdf]

The Effect of Maternal Overweight/Obesity on Serum and Breastmilk Leptin, and its Associations with Body Composition, Cardiometabolic Health Indices, and Maternal Diet: the BLOOM Study

Monika A. Zielinska-Pukos, Łukasz Kopiasz, and Jadwiga Hamulka

Table S1. Distribution of general variables of the participants among NW, OW and OB mothers.

Variable	Study group<br>M ± SD / Me (25 – 75) / *n* (%)			*p*-Value
	NW<br>*n*=20	OW<br>*n*=12	OB<br>*n*=8	
Maternal age (years)	31.9 ± 4.3	31.8 ± 3.4	34.6 ± 3.3	0.231[a]
Lactation duration (weeks)	15.7 ± 1.0	15.3 ± 1.3	15.2 ± 1.4	0.474[a]
Breastfeeding per day	11.0 (10.0 – 13.0) [a,b]	13.5 (11.5 – 15.5) [a]	10.5 (8.5 – 11.5) [b]	0.044[c]
Primiparous (*n* (%))	14 (70%)	8 (67%)	4 (50%)	0.599[d]
**Anthropometric parameters**				
Pre-pregnancy BMI (kg/m^2)	21.0 (19.9 – 22.6) [a]	28.2 (24.9 – 30.2) [b]	31.6 (30.9 – 31.7) [b]	<0.001[c]
Gestational weight gain (kg)	13.5 ± 4.1	14.1 ± 5.1	12.1 ± 9.5	0.958[c]
Current BMI (kg/m^2)	21.4 (20.4 – 22.4) [a]	26.5 (25.9 – 28.5) [b]	33.6 (30.6 – 34.9) [b]	<0.001[c]
WC (cm)	79.1 ± 5.3 [a]	90.5 ± 6.4 [b]	105.4 ± 7.4 [c]	<0.001[b]
FFM (%)	63.3 ± 4.3 [a]	57.7 ± 3.8 [b]	52.6 ± 3.2 [c]	<0.001[a]
FM (%)	34.4 ± 4.5 [a]	40.3 ± 4.0 [b]	45.9 ± 3.4 [c]	<0.001[a]
VAT (g)	284.8 (117.5 – 420.5) [a]	642.0 (439.0 – 889.0) [b]	756.5 (710.0 – 1314.0) [b]	<0.001[c]
SAT (g)	1053 (824 – 1244) [a]	1968 (1657 – 2155) [b]	2695 (2595 – 3482) [b]	<0.001[c]
**Lipid profile**				
CHOL (mg/dL)	200.0 ± 29.5	186.6 ± 39.6	212.7 ± 34.2	0.240 [a]
HDL-C (mg/dL)	81.1 ± 13.6 [a]	67.5 ± 13.6 [b]	68.3 ± 24.1 [a,b]	0.020 [b]
LDL-C (mg/dL)	107.3 ± 24.4	107.4 ± 39.5	128.0 ± 37.7	0.281 [a]
TG (mg/dL)	48.1 (44.4 – 60.9) [a]	57.0 (47.1 – 68.7) [a,b]	76.1 (59.7 – 84.3) [b]	0.033[c]
**Cardiometabolic indices**				
AIP	-0.22 (-0.27 – -0.15)	-0.08 (-0.39 – 0.08)	0.05 (-0.10 – 0.20)	0.062[c]
CMI	0.13 (0.11 – 0.15) [a]	0.19 (0.16 – 0.30) [a,b]	0.35 (0.20 – 0.43) [b]	0.004[c]
LAP	13.6 ± 5.9 [a]	21.5 ± 6.4 [b]	42.0 ± 20.5 [c]	<0.001[b]
VAI	0.52 (0.43 – 0.61) [a]	0.71 (0.58 – 1.02) [a,b]	1.14 (0.69 – 1.44) [b]	0.025[c]
**Dietary parameters**				
Energy (kcal/d) [a]	2094 ± 326	2159 ± 286	2289 ± 426	0.436[b]
Fructose (g/d) [b]	9.5 ± 4.8	10.9 ± 4.1	14.9 ± 11.0	0.271[b]
Pl-aMED	5.0 (3.5 – 6.5)	6.0 (4.5 – 6.0)	4.5 (3.5 – 6.0)	0.720[c]
**Leptin (pg/ml)**				
Serum	12406.5 ± 8819.0 [a]	17533.0 ± 7939.7 [a]	55445.4 ± 29332.5 [b]	<0.001[b]
Serum fold change	-	↑1.4x vs. NW	↑3.2x vs. OW,<br>↑4.5x vs. NW	
Breastmilk	108.9 ± 77.8 [a]	110.9 ± 62.5 [a]	685.1 ± 457.9 [b]	<0.001[b]
Breastmilk fold change	-	-	↑6.2x vs. NW&OW	

AIP, atherogenic index of plasma; BMI, body mass index; CHOL, total cholesterol; CMI, cardiometabolic index; FFM, fat-free mass; FM, fat mass; HDL-C, HDL cholesterol; LAP, lipid accumulation product; LDL-C, LDL cholesterol; M, mean; Me, median; NW, normal weight; OB, obese; OW, overweight; Pl-aMED, Polish-adapted Mediterranean Diet; SAT, subcutaneous adipose tissue; SD, standard deviation; TG, triglycerides; WC, waist circumference; VAI, visceral adiposity index; VAT, visceral adipose tissue. [a] result of the ANOVA test conducted on original data (after checking the variance heterogeneity using the Levene test) with the Tukey; [b] result of the ANOVA test conducted on log-transformed data (after checking the variance heterogeneity using the Levene test); [c] results of ANOVA Kruskal-Wallis test; [d] results of Chi2 test. The different lower case letters indicate significantly different values.

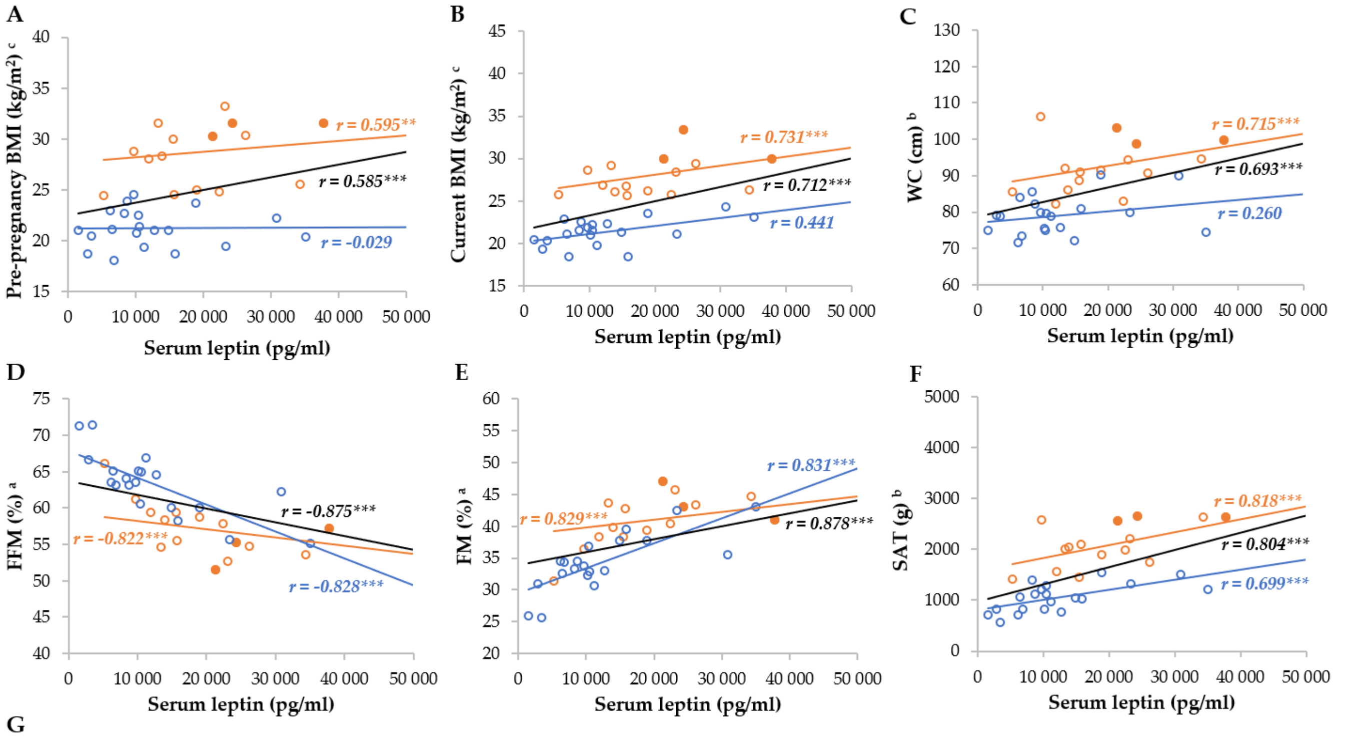

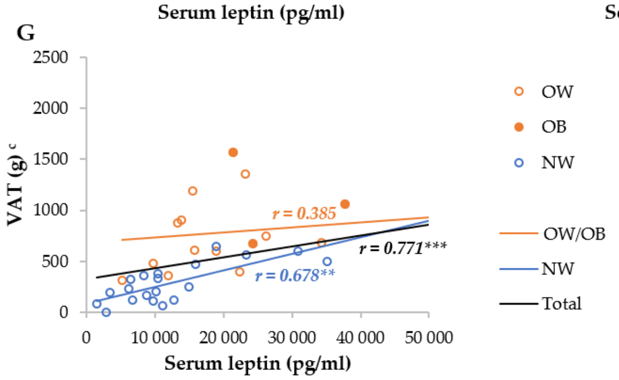

Figure S1. Correlation between serum leptin and anthropometric parameters among NW and OW/OB mothers with serum leptin not exceeding 50 000 pg/mL. BMI, body mass index; FFM, fat-free mass; FM, fat mass; NW, normal weight; OW/OB, overweight or obese; SAT, subcutaneous adipose tissue; WC, waist circumference; VAT, visceral adipose tissue. [a] result of the Pearson test conducted on original data; [b] result of the Pearson correlations conducted on log-transformed data; [c] results of the Spearman rank correlations. ** $p \leq 0.01$; *** $p \leq 0.001$.

(A) Correlation between serum leptin and pre-pregnancy BMI, (B) Correlation between serum leptin and current BMI, (C) Correlation between serum leptin and WC, (D) Correlation between serum leptin and FFM, (E) Correlation between serum leptin and FM, (F) Correlation between serum leptin and SAT, (G) Correlation between serum leptin and VAT.

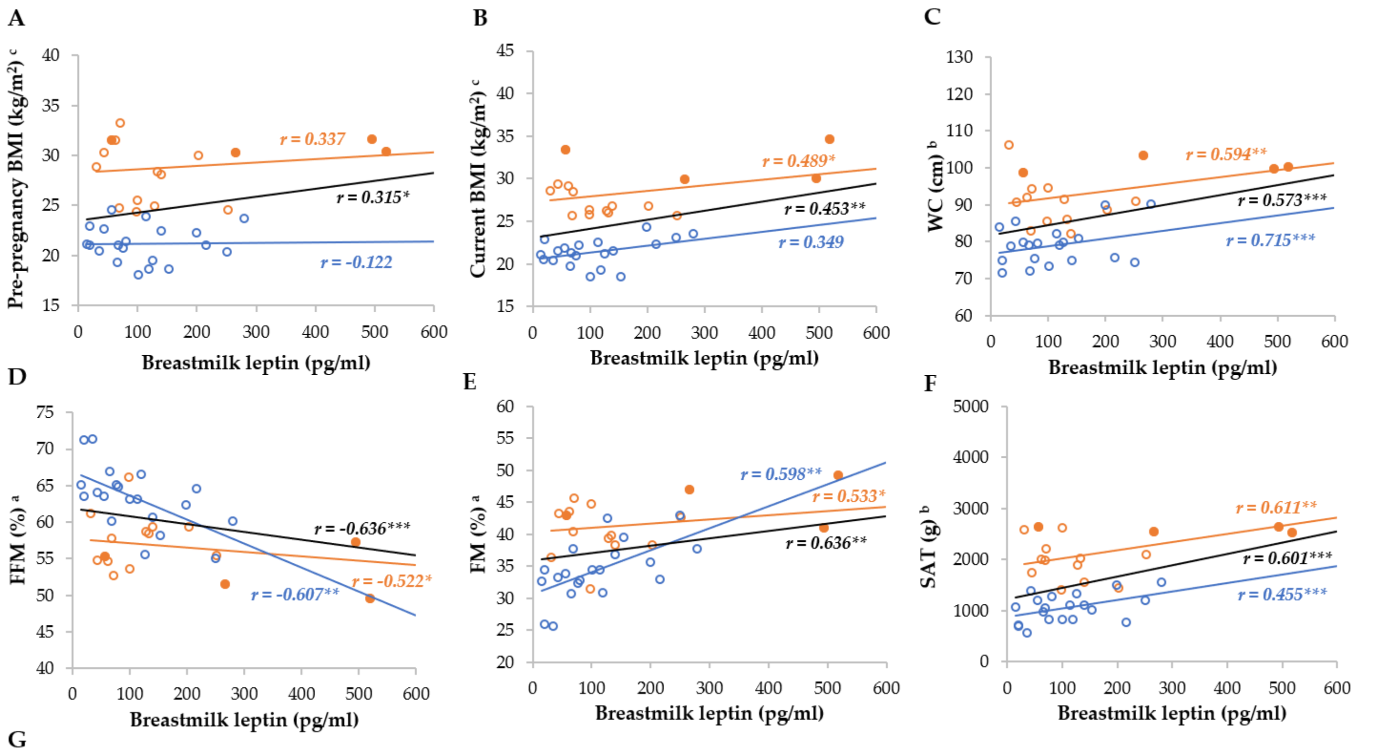

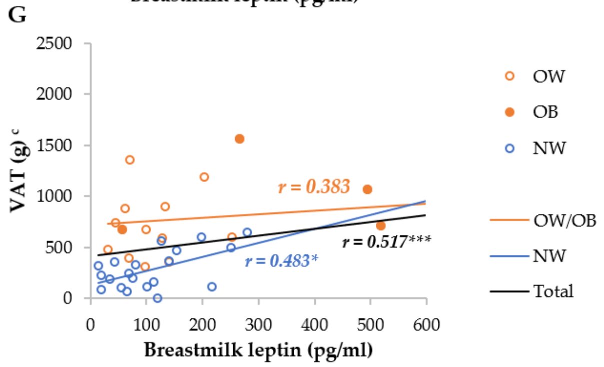

Figure S2. Correlation between breastmilk leptin and anthropometric parameters among NW and OW/OB mothers with breastmilk levels not exceeding 600 pg/ml. BMI, body mass index; FFM, fat-free mass; FM, fat mass; NW, normal weight; OW/OB, overweight or obese; SAT, subcutaneous adipose tissue; WC, waist circumference; VAT, visceral adipose tissue. [a] result of the Pearson test conducted on original data; [b] result of the Pearson correlations conducted on log-transformed data; [c] results of the Spearman rank correlations. * $p \le 0.05$; ** $p \le 0.01$; *** $p \le 0.001$.

(A) Correlation between serum leptin and pre-pregnancy BMI, (B) Correlation between serum leptin and current BMI, (C) Correlation between serum leptin and WC, (D) Correlation between serum leptin and FFM, (E) Correlation between serum leptin and FM, (F) Correlation between serum leptin and SAT, (G) Correlation between serum leptin and VAT.
